# KAP1-associated transcriptional inhibitory complex regulates C2C12 myoblasts differentiation and mitochondrial biogenesis via miR-133a repression

**DOI:** 10.1038/s41419-020-02937-5

**Published:** 2020-09-09

**Authors:** Jialing Zhang, Chaoju Hua, Yu Zhang, Peng Wei, Yaping Tu, Taotao Wei

**Affiliations:** 1 National Laboratory of Biomacromolecules, Institute of Biophysics, Chinese Academy of Sciences, 100101 Beijing, China; 2 University of Chinese Academy of Sciences, 100049 Beijing, China; 3 Department of Pharmacology and Neuroscience, Creighton University School of Medicine, Omaha, NE 68178 USA

**Keywords:** Cell biology, Developmental biology

## Abstract

The differentiation of myoblasts plays a key role in the growth of biological individuals and the reconstruction of muscle tissue. Several microRNAs are significantly upregulated during the differentiation of myoblasts and their target genes have been explored. However, the molecular mechanisms underlying the transcriptional regulation of microRNAs remain elusive. In the present study, we found that the expression of miR-133a is increased during the differentiation of C2C12 myoblasts. miR-133a mimic is sufficient to induce the biogenesis of mitochondria and differentiation of C2C12 myoblasts whereas miR-133a inhibitor abolishes cell differentiation. Using CRISPR affinity purification in situ of regulatory elements (CAPTURE) technique, we further dissected the regulatory mechanisms of miR-133a expression and found that KAP1-associated transcription complex accounts for the suppression of miR-133a in C2C12 myoblasts. Knockdown of KAP1 increased the expression of miR-133a, which contributed to the biogenesis of mitochondria and differentiation of C2C12 myoblasts. To our knowledge, this is the first study using the CAPTURE technology to identify the regulatory factors of miR-133a during cell differentiation, which may provide new ideas for understanding the precision regulatory machinery of microRNAs during different biological processes.

## Introduction

With the continuous improvement of industrialization level and the increasing labor intensity, muscle-related diseases are increasingly prevalent^1^. The differentiation of myoblasts plays a key role in both the growth of biological individuals and the reconstruction of muscle tissue; thus, dissecting the molecular mechanisms underlying myogenesis is of great importance. The differentiation of myoblasts is a complex process regulated by a number of factors^2–4^. During this process, the cell morphology changes significantly, accompanied with the rearrangement of the cytoskeleton and the reprogram of energy metabolism^5–7^. Mitochondrial biogenesis occurs during the differentiation of myoblasts into myocytes^8,9^, but the connections between mitochondrial biogenesis, myogenesis and bioenergetics have not been clearly established.

Studies have shown that several microRNAs (miRNAs) can regulate myoblasts differentiation by affecting mitochondrial energy metabolism^10,11^. For example, miR-1, a miRNA specifically induced during myogenesis^12^, efficiently enters the mitochondria where it stimulates the translation of specific mitochondrial genome-encoded transcripts, and regulates skeletal muscle differentiation by a coordinated myogenic program^13^. miR-133a is another miRNA co-transcribed with miR-1 during myogenesis^12^. We recently reported that miR-133a targets mitochondrial energy metabolism^14^. Quantitative proteomic analysis showed that the levels of oxidative phosphorylation (OXPHOS)-related proteins were significantly upregulated after transfection with miR-133a mimic, which prompted us to further explore the potential role of miR-133a in mitochondrial biogenesis. With the aid of the emerging CRISPR affinity purification in situ of regulatory elements (CAPTURE) technique^15,16^, we also investigated the transcriptional regulatory factors responsible for the upregulation of miR-133a during the differentiation of C2C12 myoblasts. Ultimately, we identified several molecules including KAP1 as the key regulator of miR-133a transcription. Our study not only links miR-133a with mitochondrial biogenesis and myoblast differentiation, but also provides a new perspective for more comprehensive studies on the regulation of miRNAs in the future.

## Results

### The differentiation of C2C12 myoblasts is accompanied with mitochondrial biogenesis

**Fig. 1:**
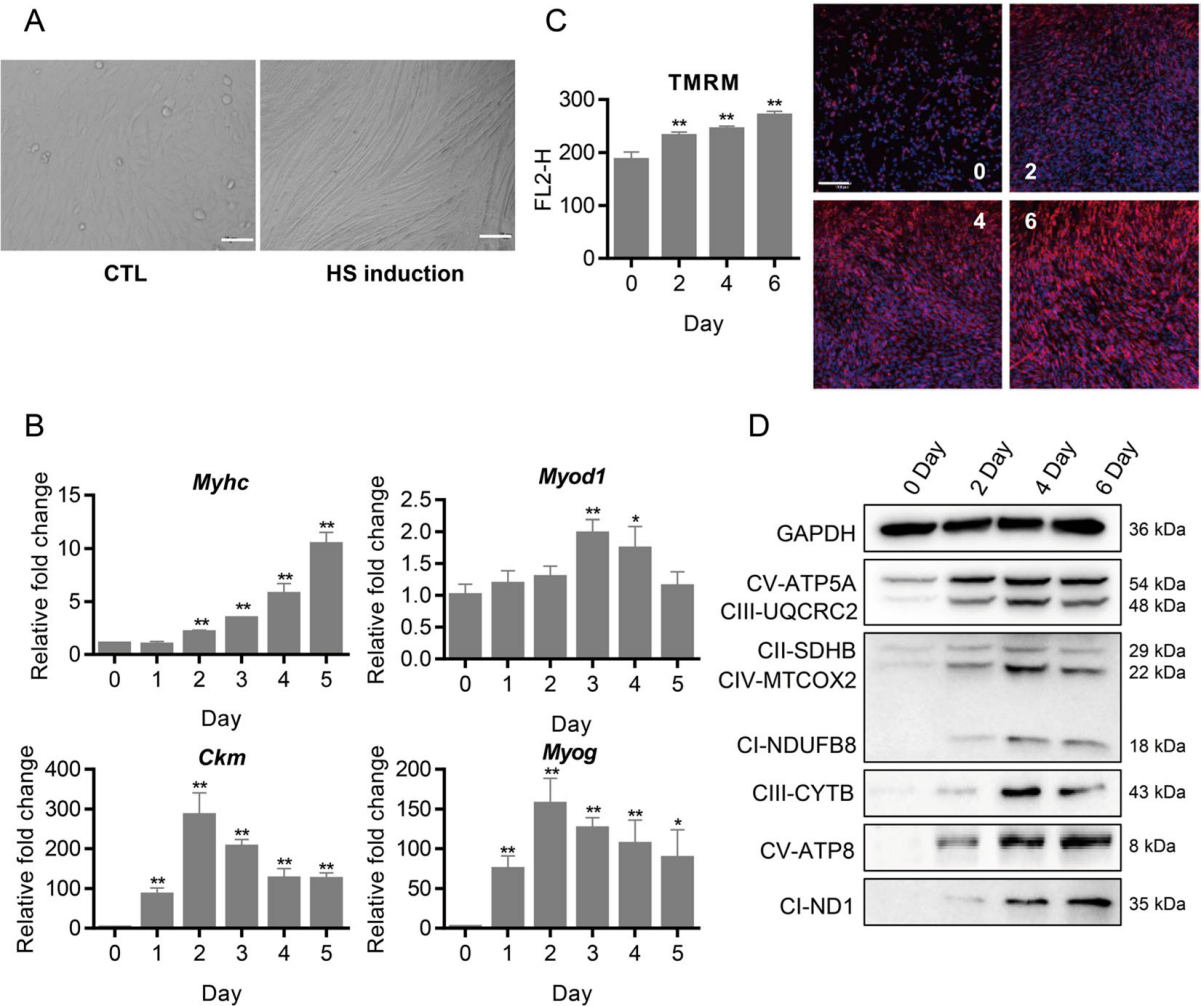
The differentiation of C2C12 myoblasts is accompanied with mitochondrial biogenesis. **a** The differentiation of C2C12 cells is confirmed by the morphological changes. C2C12 cells were induced by 2% horse serum for 6 days and images were obtained with Nikon Ti-U microscope. Scale bar: 50 μm. **b** Myogenesis-related genes were induced during the differentiation of C2C12 cells. Transcription levels of differentiation markers *Myhc*, *Myod1*, *Myog*, and *Ckm* were detected by RT-PCR during the differentiation of C2C12. Results were normalized by 18S RNA, *n* = 4. Data are presented as the mean ± SEM with *P < 0.05; **P < 0.01. **c** The differentiation of C2C12 cells is accompanied with the increase in mitochondrial transmembrane potential. C2C12 cells with different differentiation levels were stained with TMRM and Hochest and the fluorescence images were obtained with FV1000. Scale bar: 100 μm (right). The intracellular fluorescence of TMRM was also quantified by flow cytometry (left). Data are presented as the mean ± SEM (*n* = 3) with **P < 0.01. **d** The levels of mitochondrial respiratory complex subunits were increased during the differentiation of C2C12 cells. Western blot analysis of mitochondrial respiratory complex I–V subunits expression in cells with different differentiation level, data were normalized by GAPDH.

Incubation of C2C12 cells with Dulbecco’s Modified Eagle Medium (DMEM) containing 2% horse serum-induced cell differentiation, as confirmed by the morphological changes. Six days after induction, most of the cells changed morphology from spindle-shaped to tubular (Fig. 1a). Quantitative RT-PCR analysis of myogenesis-related genes *Myhc* (myosin), *Myod1* and *Myog* (transcription factors orchestrating the differentiation of muscle cells), and *Ckm* (creatine kinase) indicated time-dependent upregulation (Fig. 1b), suggesting the efficacy of horse serum-induced myoblast differentiation.

To characterize the alteration of mitochondria during myoblast differentiation, we examined the mitochondrial transmembrane potential. Confocal imaging showed that the fluorescence intensity of tetramethylrhodamine methyl ester (TMRM) increased significantly during the myoblast differentiation (Fig. 1c, right panel), as confirmed by flow cytometry (Fig. 1c, left panel), suggesting the increase in transmembrane potential of mitochondria. Following cell differentiation, the expression level of mitochondrial respiratory complex I–V subunits increased significantly (Fig. 1d). These results suggested that the differentiation of C2C12 cells was accompanied with the elevated mitochondrial activity and the biogenesis of mitochondria.

### miR-133a promotes the differentiation of C2C12 myoblasts

**Fig. 2:**
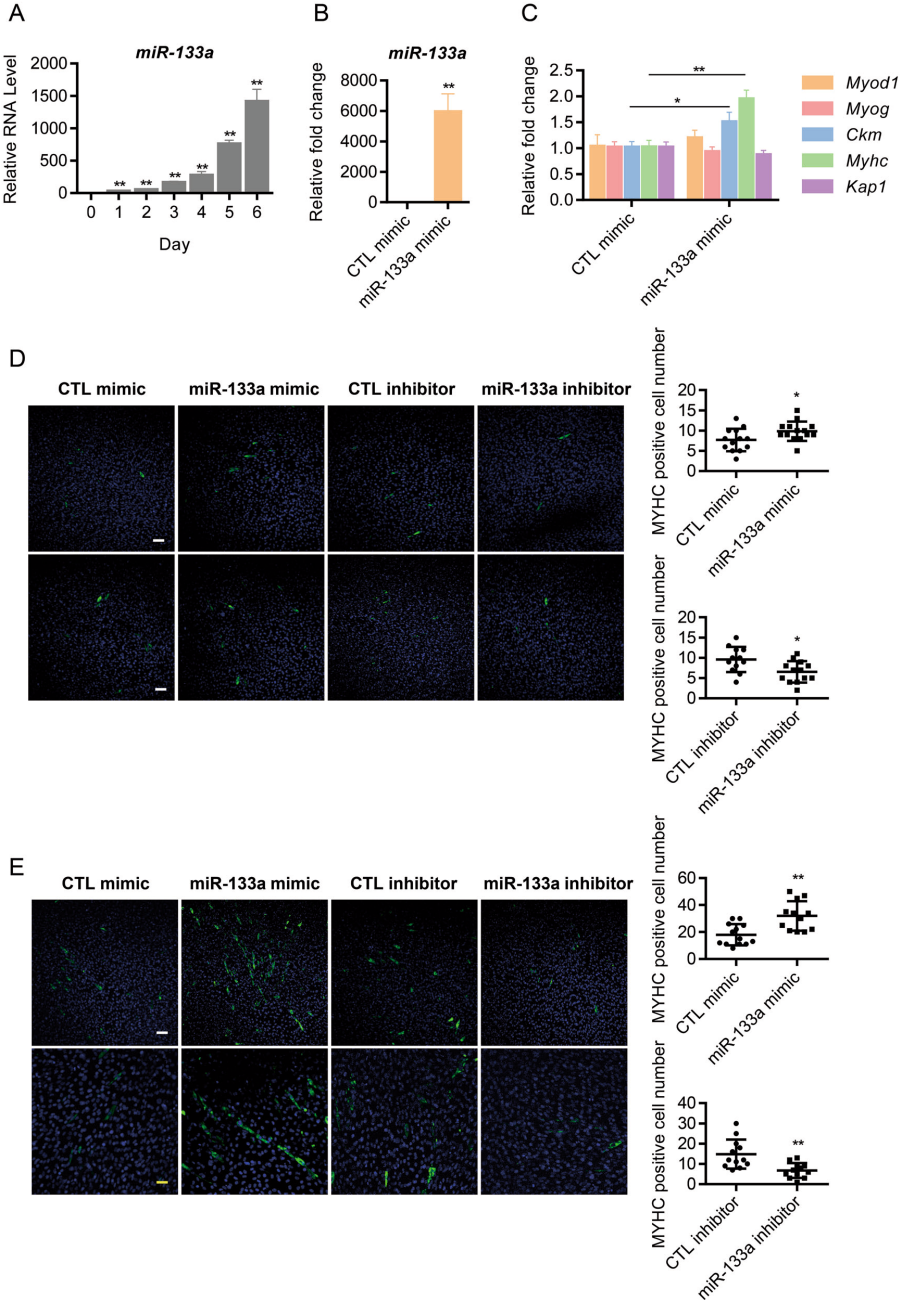
miR-133a promotes the differentiation of C2C12 myoblasts. **a** PCR quantification of miR-133a during cell differentiation. RNU6B was used as the control. Data are presented as the mean ± SEM (*n* = 5); **P < 0.01. **b** The transfection efficacy of miR-133a mimic. Intracellular miR-133a level was detected by RT-PCR after transfection with miR-133a mimic for 72 h. Data are presented as the mean ± SEM (*n* = 3); **P < 0.01. **c** RT-PCR quantification of differentiation marker *Myhc*, *Myod1*, *Myog*, and *Ckm*. Data are presented as the mean ± SEM (*n* = 4); *P < 0.05; **P < 0.01. **d** The influence of miR-133a on the number of MYHC-positive cells. C2C12 cells were transfected with miR-133a mimic or miR-133a inhibitor for 72 h and then stained for MYHC and DAPI. Fluorescence images were obtained with FV1000 microscope, scale bar = 100 μm. **e** The influence of miR-133a on the horse serum-induced differentiation of C2C12 cells. Cells were transfected with miR-133a mimic or inhibitor for 72 h and then induced by 2% horse serum for 4 days, stained for MYHC and DAPI, and fluorescence images were obtained with FV1000. Scale bar: 100 μm (up) and 50 μm (down). Data are presented as the mean ± SEM (*n* ≥ 12), *P < 0.05, **P < 0.01.

We quantified the dynamics of miR-133a level during the differentiation of C2C12 myoblasts. As shown in Fig. 2a, the level of miR-133a was increased by ~1500-fold on the 6th day of differentiation in comparison with undifferentiated myoblasts. The level of miR-1 that is co*-*transcribed with miR-133a was also increased during the differentiation of C2C12 myoblasts (Fig. S1A).

We used miR-133a mimic and inhibitor to determine the role of upregulated miR-133a in myoblast differentiation. C2C12 myoblasts were transfected with miR-133a mimic for 72 h and the transfection efficacy was confirmed by quantitative PCR (Fig. 2b). Upon miR-133a mimic transfection, the expression of *Ckm* and *Myhc*, two marker genes associated with myoblast differentiation, was upregulated (Fig. 2c); however, the expression of *Myod1* and *Myog*, two transitional factors orchestrating myogenesis, was not affected.

We further confirmed the effects of miR-133a on myoblast differentiation by quantifying the MYHC-positive cells. Results shown in Fig. 2d indicated that exposure of C2C12 cells to miR-133a mimic caused a significant increase in the number of MYHC-positive cells. In contrast, miR-133a inhibitor significantly decreased the percentage of MYHC-positive cells.

Next, we investigated the effects of miR-133a on horse serum-induced myoblast differentiation. C2C12 cells were transfected with either miR-133a mimic or miR-133a inhibitor, and then were exposed to 2% horse serum for 4 days. As shown in Fig. 2e, miR-133a mimic synergistically increased the number of horse serum-induced MYHC-positive cells whereas miR-133a inhibitor significantly decreased the number of horse serum-induced MYHC-positive cells. These data suggest that upregulated miR-133a contributes to myoblast differentiation.

### Identification of regulatory molecules associated with the upstream region of miR-133a

**Fig. 3:**
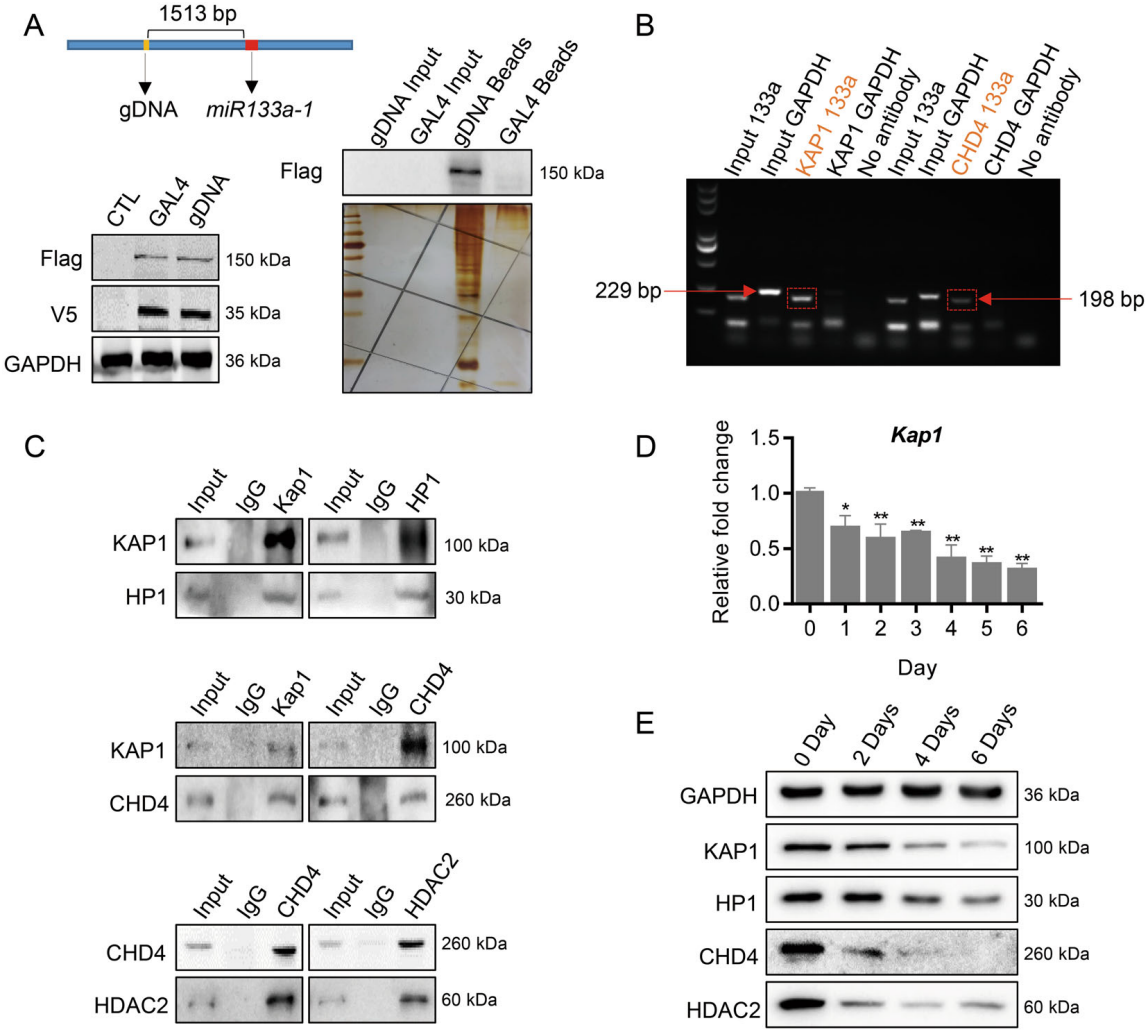
Identification of regulatory molecules associated with the upstream region of miR-133a. **a** Design and construction of the CAPTURE system. gDNA was designed according to the miR-133a1 locus (up). C2C12 cells were transfected with gDNA, dCas9, and BirA by lentivirus and were sorted by flow cytometer. Proteins extracted by streptavidin beads were detected by western blot (left) and silver staining (right), respectively. The expression of dCas9 (Flag) and BirA (V5) were detected in transfected C2C12 cells. **b** ChIP-PCR indicates the binding of KAP1 and CHD4 proteins at the upstream region of miR-133a gene. The ChIP DNA fragments were pulled down by KAP1 antibody or CHD4 antibody, respectively, and were amplified by PCR using primers for the upstream region of miR-133a or the housekeeping gene GAPDH. The miR-133a fragment (198 bp; highlighted with red frame) could be detected in ChIP DNA fragments pulled down by KAP1 or CHD4 antibody, suggesting the binding of KAP1 and CHD4 proteins at the upstream region of miR-133a1 gene. No GAPDH fragment (229 bp) could be detected in KAP1 or CHD4 groups. **c** Co-IP experiments confirmed the protein-protein interactions between KAP1 and HP1, KAP1, and CHD4, CHD4, and HDAC2 in C2C12 cells. **d** Decrease in *Kap1* expression during the differentiation of C2C12 cells. The transcription level of *Kap1* in C2C12 cells during differentiation was detected by quantitative RT-PCR. The result was normalized by 18 S RNA. Data are presented as the mean ± SEM (*n* = 4); *P < 0.05; **P < 0.01. **e** Decrease in KAP1-associated complex expression during the differentiation of C2C12 cells. The expression profiles of KAP1, HP1, CHD4, and HDAC2 were detected by western blot. GAPDH was used as an internal control.

Given the fact that upregulated miR-133a is involved in the differentiation of C2C12 myoblasts, our next aim is to investigate which factors are responsible for the regulation of miR-133a transcription during myoblast differentiation. We used the CRISPR affinity purification in situ of regulatory elements (CAPTURE) technique that could pull out the particular sequence of chromatin and identify transcription regulators bound to that segment. This method is based on the CRISPR-Cas9 technology, in which nuclease-deficient Cas9 (dCas9) protein binds on the upstream region of miR-133a1 gene under the guidance of gDNA^17,18^. BirA biotinylates dCas9 protein, thus dCas9 could be pulled down by streptavidin magnetic beads. At the same time, other proteins associated with this segment would also be pulled down, and be detected by mass spectrometry. We successfully obtained C2C12 cell lines that express both dCas9 and BirA stably, among which GAL4 cell line did not express gDNA. Results of silver staining and western blot showed the dCas9 protein could only bind to chromatin and be pulled in cells expressing gDNA, whereas almost no proteins were pulled down in the control group (Fig. 3a).

Using this experimental system and with the aid of mass spectrometry, we identified several transcription regulators associated with the upstream region of miR-133a1 gene, including KAP1, CHD4 (the core component of the NuRD complex), HDAC2 (an acetyltransferase), and HP1. Results of ChIP-PCR indicated that KAP1 and CHD4 indeed bound to the upstream region of miR-133a1 gene (Fig. 3b). In addition, the protein-protein interactions between KAP1 with HP1, KAP1 with CHD4, and CHD4 with HDAC2 were confirmed in C2C12 cells (Fig. 3c).

We analyzed the expression of these genes during the differentiation of C2C12 cells and found that both KAP1 mRNA (Fig. 3d) and protein (Fig. 3e) were downregulated gradually. The expression of HP1, CHD4, and HDAC2 proteins were also downregulated gradually during the differentiation of C2C12 cells (Fig. 3e).

### KAP1 complex regulates C2C12 myoblasts differentiation via miR-133a repression

KAP1 acts as a scaffold protein and forms a dynamic transcriptional regulatory system in which corepressor and coactivator complexes are superimposed, and plays important roles in the regulation of various physiological processes^19–21^. There is also evidence that KAP1 modulates C2C12 myoblasts differentiation by activating transcription factors MYOD and Mef2^22^. Therefore, we focused our research on KAP1 and investigated the effects of alterations of KAP1 expression on myoblast differentiation of C2C12 cells.

**Fig. 4:**
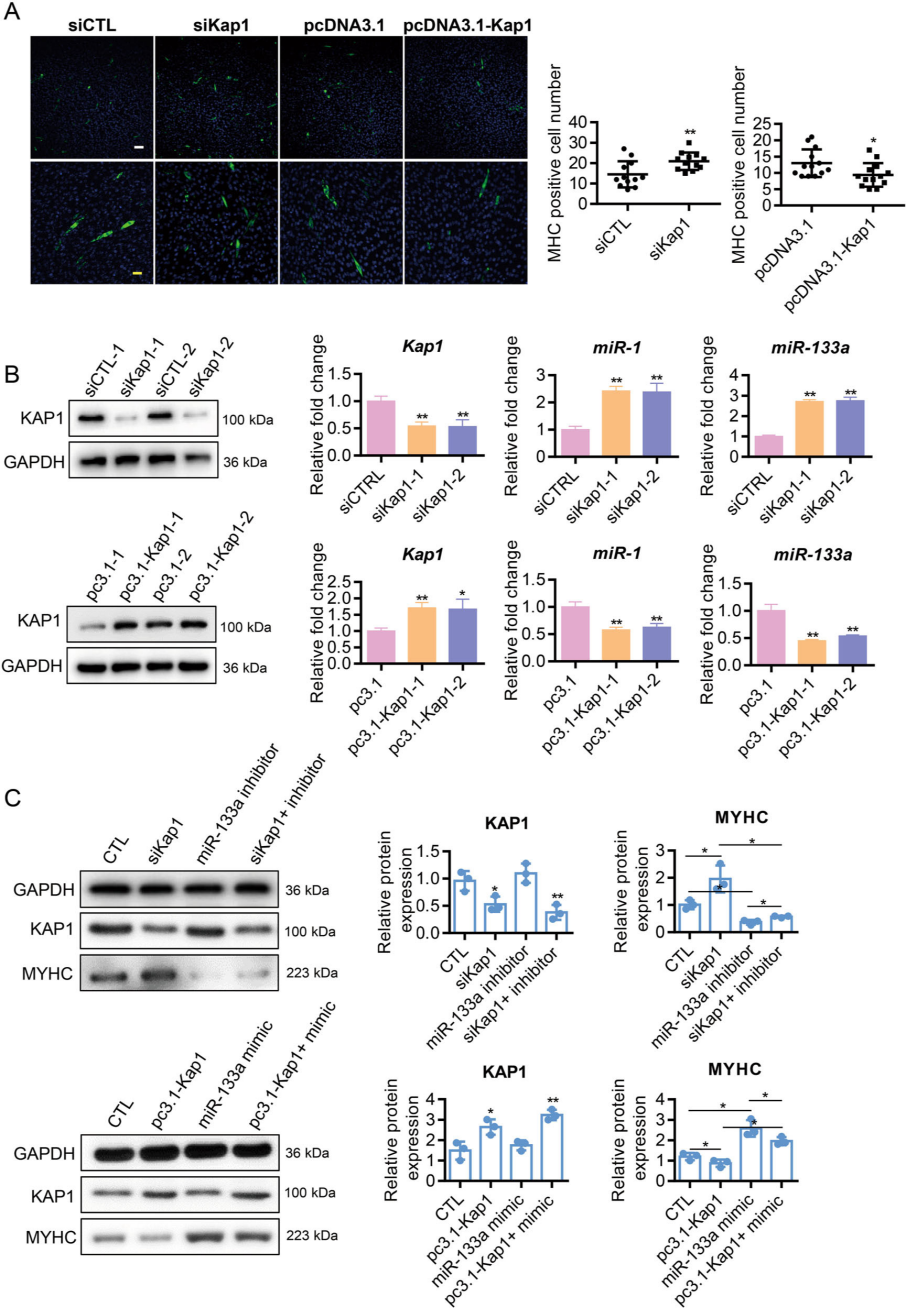
KAP1 complex regulates C2C12 myoblasts differentiation via miR-133a repression. **a** KAP1 regulates the differentiation of C2C12 cells. The expression of Kap1 in C2C12 cells was manipulated with siRNA or pcDNA3.1-Kap1 plasmid transfection, respectively, and then cells were induced for differentiation by 2% horse serum for 4 days, stained for MYHC and DAPI, and fluorescence images were obtained with FV1000 microscope. Scale bar: 100 μm (up) and 50 μm (down). Data are presented as the mean ± SEM (*n* ≥ 12); *P < 0.05; **P < 0.01. **b** KAP1 regulates miR-1 and miR-133a expression. The expression of KAP1 in C2C12 cells was manipulated with siRNA or pcDNA3.1-Kap1 plasmid transfection and the efficacy was detected by western blot and quantitative RT-PCR. The influence of KAP1 on miR-1 and miR-133a expression was detected by quantitative RT-PCR. Data are mean ± SEM (*n* = 4); *P < 0.05; **P < 0.01. **c** KAP1 regulates C2C12 myoblasts differentiation via miR-133a-related mechanisms. The expression of KAP1 in C2C12 cells was manipulated with siRNA or pcDNA3.1-Kap1 plasmid transfection. Then cell differentiation was induced by 2% horse serum in the presence of miR-133a inhibitor/mimic. The expression levels of KAP1 and MYHC were detected by western blot, and signal intensity was normalized with GAPDH and shown as scatter plot with individual data point graph with significance. Data are presented as the mean ± SEM (n = 3), *P < 0.05; **P < 0.01.

Knockdown of KAP1 significantly promoted cell differentiation whereas overexpression of KAP1 inhibited cell differentiation, as indicated by the significant changes in MYHC expression (Fig. 4a). We also found that knockdown of KAP1 resulted in a significant increase of miR-133a in C2C12 cells whereas overexpression of KAP1 caused down-regulation of miR-133a (Fig. 4b). Interestingly, miR-1, which is co-transcribed with miR-133a, was also negatively regulated by KAP1 (Fig. 4b).

To ascertain the role of miR-133a in KAP1-dependent C2C12 cell differentiation, we manipulated the expression of KAP1 in C2C12 cells and treated cells with mimics or inhibitors of miR-133a. We transfected cells with siKap1 with or without miR-133a inhibitor for 72 h. Cells were then exposed to horse serum to initiate cell differentiate. Results of western blots indicated that knockdown of KAP1 accelerated cell differentiation in C2C12 cells, which could be partially abolished by miR-133a inhibitor (Fig. 4c). Similarly, overexpression of KAP1 suppressed cell differentiation in C2C12 cells, which could be partially reversed by miR-133a mimic (Fig. 4c), suggesting that KAP1 regulates C2C12 cell differentiation, at least in part, via miR-133a-related mechanisms.

### miR-133a promotes mitochondrial biogenesis in C2C12 myoblasts

**Fig. 5:**
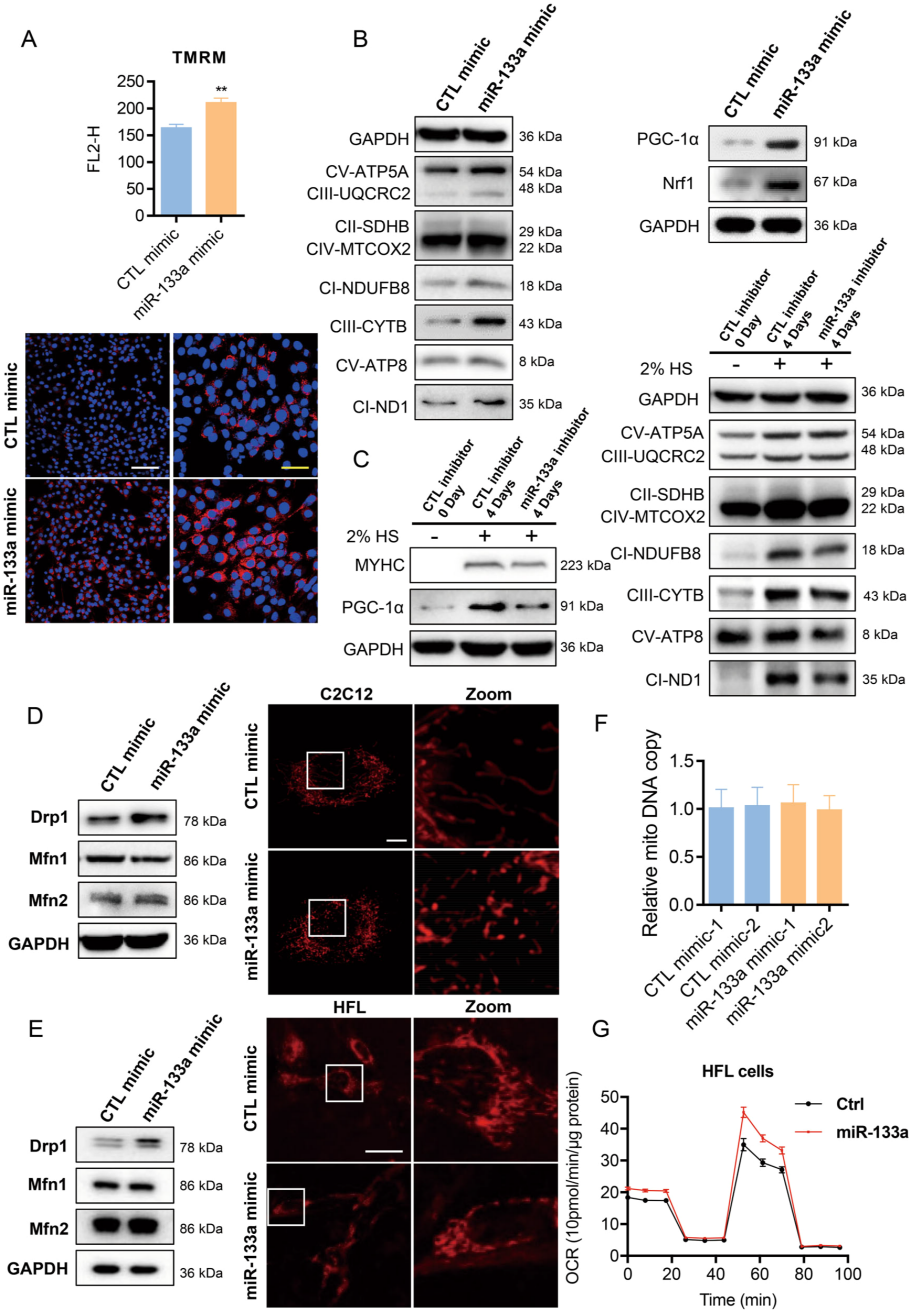
miR-133a promotes mitochondrial biogenesis in C2C12 myoblasts. **a** miR-133a increases the mitochondrial transmembrane potential in C2C12 cells. C2C12 cells were transfected with miR-133a mimic for 72 h and stained with TMRM and Hochest. Intracellular TMRM fluorescence was measured by both FV1000 microscope (down; scale bar: 100 μm and 50 μm) and flow cytometry (up). Data are presented as the mean ± SEM (*n* = 3), **P < 0.01. **b** miR-133a upregulates the expression of mitochondrial complex subunits via PGC-1α/Nrf1 axis in C2C12 cells. C2C12 cells were transfected with miR-133a mimic for 72 h and the expression levels of mitochondrial respiratory complex subunits, PGC-1α and Nrf1 were detected by western blot. **c** Inhibition of miR-133a down regulates the expression of PGC-1α and mitochondrial complex subunits in differentiating C2C12 cells. C2C12 cells were transfected with miR-133a inhibitor for 72 h and induced differentiation for 4 days, then the expression levels of mitochondrial respiratory complex subunits, MYHC and PGC-1α were detected by western blot. **d** miR-133a induces mitochondrial fission in C2C12 cells. C2C12 cells were transfected with miR-133a mimic for 72 h and the expression levels of mitochondrial fusion proteins Mfn1, Mfn2 and mitochondrial fission protein Drp1 were detected by western blot. Mitochondria was stained with MitoTracker Red, and the morphology was captured by SIM microscopy (right). Scale bar: 5 μm. **e** miR-133a induces mitochondrial fission in human lung fibroblasts (HFL). HFL cells were transfected with miR-133a mimic for 72 h and the expression levels of mitochondrial fusion proteins Mfn1, Mfn2, and mitochondrial fission protein Drp1 were detected by western blot. Mitochondria was stained with MitoTracker Red, and the morphology was captured by FV1000 microscopy (right). Scale bar: 50 μm. **f** miR-133a shows no impact on mitochondrial DNA copy number. C2C12 cells were transfected with miR-133a mimic for 72 h and mitochondrial DNA copy number was determined by RT-PCR. Data are presented as the mean ± SEM (*n* = 4). **g** miR-133a enhances mitochondrial respiratory. HFL cells were seeded into XF24 cell-culture plates, transfected with or without miR-133a mimic for 72 h. The real-time detection of OCR could be monitored using the Seahorse XF24–3 extracellular flux analyzer. Data represent the mean ± SEM (*n* = 5).

The above data indicated that miR-133a is upregulated during the differentiation of C2C12 cells via a KAP1-related regulation pathway. Since our recent work demonstrated that miR-133a induces the upregulation of OXPHOS genes, we further investigated the effects of miR-133a on the biogenesis and function of mitochondria. Transfection of miR-133a mimic caused a marked increase in the mitochondrial transmembrane potential in C2C12 cells (Fig. 5a), similar to the observation in horse serum-treated (Fig. 1c) or miR-1-treated C2C12 cells (Fig. S1B). Next, we examined the expression level of PGC-1α, a master regulator of mitochondrial biogenesis^8,23^. PGC-1α and its effector protein Nrf1, as well as several subunits of the mitochondrial complexes (ND1 and NDUFB8 of complex I; CYTB and UQCRC2 of complex III; COX2 of complex IV; ATP5A and ATP8 of complex V), were upregulated by miR-133a mimic (Fig. 5b). On the contrary, the inhibitor of miR-133a downregulated the expression of PGC-1α and some subunits of the mitochondrial respiratory complexes (Fig. 5c). Western blots signal intensities of Fig. 5b, c were further quantified and shown as scatter plot with individual data point graph with significance (Figs. S2 and S3).

The impact of miR-133a mimic on mitochondrial dynamics was also investigated. Transfection of miR-133a mimic caused mitochondrial fission in C2C12 cells (Fig. 5d). This process was accompanied with the upregulation of mitochondrial dynamin-related protein 1 (Drp1) that orchestrates the fission of mitochondrial network. Mfn1 and Mfn2, two key players involved in mitochondrial fusion, were not affected by miR-133a mimic. Similar results were also obtained in human lung fibroblasts (HFL; Fig. 5e), suggesting a pivotal role of miR-133a in mitochondrial biogenesis and dynamics. However, the copy number of mtDNA was not affected by miR-133a (Fig. 5f).

We further studied the effects of miR-133a on mitochondrial OXPHOS. In situ analysis of oxygen consumption by Seahorse XF24 confirmed that miR-133a mimic elevated both the basal and the maximal respiratory rate in HFL cells (Fig. 5g), suggesting a significant elevation of the mitochondrial respiration upon miR-133a mimic treatment.

## Discussion

We hereby provide direct evidences that miR-133a is involved in myoblast differentiation by inducing mitochondrial biogenesis and enhancing mitochondrial respiratory, which is crucial for the generation of ATP and other molecules needed for differentiation.

Myoblasts are stem cell-like muscle precursor cells that have significant therapeutic potential to treat muscle dystrophy and severe skeletal muscle trauma. Upon activation, myoblasts recapitulate embryonic muscle development, which involves proliferation, cell cycle withdrawal, and subsequent differentiation to fuse into multinucleated syncytial myotubes^3,24^. Different from myoblasts which rely primarily on glycolysis, the metabolically active myotubes rely on mitochondrial OXPHOS^25^. To meet their high energetic demand, mitochondria undergo extensive remodeling in their morphology, activity, and content during this highly orchestrated myogenic process^26,27^. However, the events that set the stage for mitochondrial biogenesis during myogenic differentiation remain elusive. Recently, myogenic miRNAs are regarded as key players for mitochondrial biogenesis and metabolic reprogram.

miRNA is a class of endogenous, highly conserved, noncoding single-stranded RNA molecules with length of 20–24 nucleotides, which is mainly involved in the regulation of posttranscriptional gene expression^28–30^ and plays variety of important regulatory roles in vivo, such as cell proliferation, cell differentiation, stress adaption, and cell death^31–33^. By using C2C12 myoblasts as a cell model, miR-1 was found to be upregulated during C2C12 differentiation, which promotes myogenesis by targeting histone deacetylase 4 (HDAC4)^12^. Further investigation indicated that miR-1 efficiently enters the mitochondria by interacting with Ago2 and thus coordinates the regulatory networks in both the cytoplasm and the mitochondria during and after muscle differentiation^13^. Recently, 15 additional myogenic miRNAs were further identified during C2C12 differentiation, by targeting mitochondrial biogenesis and metabolic reprogram^10,11^.

miR-1 and miR-133a are clustered on the same chromosomal loci and transcribed together during development^34^, and they were reported to have distinct cellular effects, probably by regulating different target genes. miR-1 promotes myogenesis by targeting HDAC4 and other mitochondrial targets, while miR-133a enhances myoblast proliferation and suppresses differentiation by repressing serum response factor (SRF)^12^. However, our present investigation indicates that prolonged exposure of C2C12 myoblasts to mimic of miR-133a induces differentiation, as evidenced by the induction of *Myhc* expression and the increase of MYHC-positive cells. Mimic of miR-133a also accelerates the differentiation of C2C12 cells induced by horse serum, whereas miR-133a inhibitor blocks differentiation of C2C12 cells.

We next set up a CRISPR affinity purification in situ of regulatory elements (CAPTURE) system to investigate the upstream factors involved in miR-133a transcription during C2C12 differentiation. Upon in situ biotinylation of dCas9 by the biotin ligase BirA in mammalian cells, the genomic locus-associated macromolecules could be isolated by high-affinity streptavidin purification. The purified protein, RNA, and DNA complexes could be identified and analyzed by mass spectrometry-based proteomics and high-throughput sequencing, respectively. At the same time, the dCas9/gDNA system can be manipulated by altering gDNA sequences or combinations, thus allowing for medium- to high-throughput analysis of chromatin interactions^15^. With the aid of CAPTURE technique, we dissected the regulatory mechanisms of miR-133a and found that KAP1-associated transcription regulatory complex accounts for the suppression of miR-133a. KAP1 is a scaffold protein which recruits both transcription coactivators and corepressors, and modulates gene expression in a combinatorial and signal-dependent fashion^19–21^. During the initiation stage of skeletal muscle differentiation, MSK1-mediated phosphorylation of KAP1 releases the corepressors G9a and HDAC1 from the scaffold, and thus activates the MYOD/MEF2-dependent muscle gene expression program^22^. Our CAPTURE experiments indicated that KAP1 recruits CHD4, HDAC2, and HP1 protein in undifferentiated C2C12 myoblasts, forms a transcriptional regulatory complex and disturbs the binding of RNA polymerase with chromatin, thus inhibits the transcription of miR-1 and miR-133a. Upon horse serum-induced myoblast differentiation, the protein levels of KAP1, CHD4, HDAC2, and HP1 were downregulated, which results in the opening of chromatin structures. These changes are more conducive to the binding of RNA polymerase, thereby activate the transcription of miR-1 and miR-133a and accelerate the differentiation of myoblasts. Since KAP1 knockdown-accelerated C2C12 cell differentiation could be abolished by inhibitors of miR-133a, while KAP1 overexpression-suppressed C2C12 cell differentiation could be restored by mimics of miR-133a, we could conclude that KAP1 complex regulates C2C12 differentiation, at least in part, via miR-133a-related mechanisms. KAP1-regulated miR-1 might also play important roles during C2C12 differentiation.

Next, we seek clues linking miR-133a with mitochondrial biogenesis. Mimics of miR-133a induced the expression of PGC-1α and its effector gene Nrf1, indicating the activation of the PGC-1a/Nrf1 axis, which is crucial for the mitochondrial biogenesis. We found that miR-133a not only upregulated the nuclear DNA-encoded subunits, but also increased the mitochondrial DNA-encoded respiratory complexes subunits, suggesting a mitochondrial transcription-enhancing effects, similar with miR-1 (Fig. S1C).

We also observed that miR-133a induced mitochondria fission in C2C12 myoblasts, probably via the upregulation of Drp1. Drp1 is the mediator of mitochondrial fission and is essential for skeletal muscle differentiation^27,35,36^. It has been well addressed that after C2C12 is induced to differentiate, the cell metabolism will change from highly glycolytic to mitochondrial OXPHOS. This phenomenon requires the reconstruction of the mitochondrial network, which mainly includes two processes: mitochondrial clearance and biogenesis. The process of mitochondrial clearance is mainly mediated by upregulation of the mitochondrial fission protein Drp1 and the mitochondrial autophagy receptor protein SQSTM1^27,35,36^. Thus, miR-133a might also involve in myoblast differentiation via regulation of mitochondrial dynamics, although the precious molecular mechanisms remain to be explored.

In conclusion, we hereby provided direct evidence that miR-133a is one of the key factors during the myogenesis. We uncovered the effects of KAP1-associated regulatory complex in controlling miR-133a expression during myoblast differentiation. We also linked miR-133a with the biogenesis and dynamics of mitochondria during myogenesis. The application of CAPTURE technology to catch the specific genomic loci may provide new ideas for understanding the precision regulatory machinery of miRNAs during different biological processes.

## Materials and methods

### Cell culture and induction of differentiation

C2C12 and HEK-293t cells were purchased from ATCC (Manassas, VA, USA) and cultured in DMEM (Hyclone, Logan, UT, USA) supplemented with 10% fetal bovine serum (Gibco, Grand Island, NY, USA) in 37 °C incubator with 5% CO_2_. Human primary lung fibroblast cells (HFL) were established by Dr. Reynold Panettieri’s laboratory and cultured in DMEM/ F-12 (Gibco) supplemented with 10% fetal bovine serum^37^. For induction of myoblast differentiation, cell growth medium was replaced with DMEM containing 2% horse serum (Hyclone) when C2C12 cell confluence reached 95%.

### Quantitative RT-PCR

RNA was extracted with TRIzol Reagent (Thermo Fisher, Waltham, MA, USA) and cDNA was acquired by reverse transcription using HiFiScript Kit (CWBiotech, Beijing, China). mRNA and miR-133a level were detected by UltraSYBR Mixture (CWBiotech) or GoldStar TaqMan Mixture (CWBiotech), and normalized by 18S and *RUN6B*, respectively. PCR primers were listed in Supplementary Information 2.

### Detection of mitochondiral DNA copy number

Total DNA was extracted by the Universal Genomic DNA Kit (CWBiotech). Mitochondrial gene *Cox1* and nuclear gene *Pecam* were selected as the representative genes of mitochondrial and nuclear DNA, respectively. Quantitative PCR was used to detect the copy number changes of *Cox1*.

### Fluorescence microscopy and flow cytometry

For estimation of mitochondrial transmembrane potential, C2C12 cells cultured on glass coverslips were stained with 100 nM TMRM (Invitrogen, Eugene, OR, USA) at 37 °C for 20 min. Microscopic images were obtained by the Olympus FV1000 confocal laser scanning microscope (Olympus, Osaka, Japan), and Imaris X64 9.2.1 software was used for image processing. Intracellular TMRM intensity was also quantified with a BD FACSCalibur flow cytometer (BD Bioscience, Mountain View, CA, USA), and data were processed by FlowJo 10 software. Detailed information about probes and methods could be found in Supplementary Information 3.

### Acquisition of monoclonal cell line

The plasmids of pEF1a-FB-dCas9-puro and pEF1a-BirA-V5-neo were generous gifts from Jian Xu laboratory (Children’s Medical Center Research Institute, Department of Pediatrics, University of Texas Southwestern Medical Center, Dallas, TX, USA). C2C12 cells were transfected with pEF1a-FB-dCas9-puro and pEF1a-BirA-V5-neo plasmids with Lipofectamine 3000 reagent simultaneously. After three days of selection with 1.0 μM puromycin (Beyotime, Shanghai, China) and 400 nM G418 (Inalco, Beijing, China), cells were diluted into 96-well plates with one cell in each well. Western blot was used to detect the expression of BirA and dCas9. The monoclonal cell lines expressing both BirA and dCas9 were kept for subsequent experiments.

### gDNA design

The sequence of murine miR-133a was downloaded from the National Center for Biotechnology Information and previously published reference^34^. Gene Transcription Regulation Database was used to select the region with abundant transcription factors in the noncoding region, and about 250 bp length of sequence was extracted. gDNA was designed according to information about the miR-133a1 locus^34^ by the website (crispr.mit.edu), and the sequence with highest score was chosen. A 166 bp length of fragment including gDNA was synthesized by Sangon Biotech (Shanghai, China). gDNA: GGGACAGCTGATCTAAGTGC Sequence for synthesis: sgtatcccttggagaaccaccttgttggGGGACAGCTGATCTAAGTGCgtttaagagctatgctggaaacagcatagcaagtttaaataaggctagtccgttatcaacttgaaaaagtggcaccgagtcggtgctttttttctcgagt actaggatccattaggcgg.

### Plasmid construction and transfection

pMDLg/pRRE, pRSV-Rev, pMD2.G, pSLQ1651-sgRNA(F+E)-sgGAL4 plasmids were obtained from Addgene; vector of pcDNA3.1-Kap1 was purchased from YouBio (Changsha, China). *BstX*I and *Xho*I endonucleases cut the synthesized fragment and GAL4 plasmid, then T4 DNA ligase linked the plasmid and fragment to obtain GAL4-gDNA vector. Plasmid sequencing was performed by Sangon Biotech (Shanghai, China). Lipofectamine^®^ 3000 reagent (Thermo Fisher, San Jose, CA, USA) was used for plasmid transfection.

### Lentivirus packing

Plasmids pMDLg/pRRE, pRSV-Rev, pMD2.G, and pSLQ1651-sgRNA(F+E)-sgGAL4 were transfected into HEK-293t cells simultaneously for 72 h, then the cell supernatant was collected, filtered with 0.22 μm filter membrane, and added to the C2C12 cells. Another 72 h later, the cells expressing mCherry were sorted by flow cytometer to obtain cell lines that express gDNA, dCas9 and BirA at the same time.

### dCas9 pull-down

Cells expressing gDNA and in control (transfected with GAL4 plasmid) groups were all expanded to 150 mm culture dishes, crosslinked with 2% formaldehyde, and quenched with 0.25 M glycine^16^. Cells were treated with cell lysis buffer and nuclear lysis buffer to isolate chromatin. Then the separated chromatin was washed with the mixture of 8 M urea, re-suspended in the IP-binding buffer, and sonicated into ~500 bp length of segments. Hydrophilic streptavidin magnetic beads (NEB, Ipswich, MA, USA) were added into the IP-binding buffer and rotated overnight at 4 °C to isolate dCas9 protein. Isolated proteins were subjected to western blot analysis and SDS-PAGE. Proteins in SDS-PAGE gels were visualized after silver staining^15,16^.

### LC–MS/MS mass spectrometry

Silver-dyed gels were decolorized and reduced with DTT. After iodoacetyl alkylation, trypsin was added to the silver-dyed strips. The trypsin-digested peptides were extracted overnight with 60% acetonitrile, followed by analyzing with liquid chromatography-linear ion trap mass spectrometer (nanoLC-LTQ-Orbitrap XL, Thermo Fischer). Data analysis was performed by Proteome Discoverer (version 1.4.0.288, Thermo Fischer) software, and MS2 spectra were searched by the SEQUEST search engine.

### ChIP-PCR

ChIP-PCR was performed to ascertain the binding of protein with DNA. In brief, C2C12 cells with 90% confluency were lysed by using a ChIP Kit (Beyotime) to isolate chromatin. Chromatin was broken into segments, incubated with antibodies against KAP1 and CHD4, and then with Protein A-agarose beads (CWBIO). ChIP samples were purified with PCR/DNA purification Kit (Beyotime), and amplified by PCR, with primers targeting *miR-133a* or *Gapdh*. The existence of miR-133a fragment (198 bp) indicates the binding with *miR-133a* gene.

### Immunoprecipitation

C2C12 cells with 90% confluency were lysed with CO-IP buffer (50 mM Tris-HCl, 150 mM NaCl, 1% Triton X-100, 1× protease inhibitor) for 20 min on ice. After centrifuged at 12,000 × rpm for 20 min, the supernatant was rotated with antibody and Protein A-agarose beads at 4 °C for 14 h. Isolated beads were washed for three times and analyzed by western blot.

### Western blot

The protein concentration was quantified with Pierce BCA Kit (Rockford, IL, USA). Western blot analysis was performed as we described previously^38^. Antibodies are listed in Supplementary Information 3.

### Data analysis

All data were represented as the mean ± SEM. The experiments were performed at least three times. The Student’s unpaired *t* test was used for analysis between two groups. *P* < 0.05 indicates the distinction is significant.

## Supplementary information

Supplementary Information 1

Supplementary Information 2

Supplementary Information 3
