# Correction: KAP1-associated transcriptional inhibitory complex regulates C2C12 myoblasts differentiation and mitochondrial biogenesis via miR-133a repression

**DOI:** 10.1038/s41419-026-08844-5

**Published:** 2026-06-11

**Authors:** Jialing Zhang, Chaoju Hua, Yu Zhang, Peng Wei, Yaping Tu, Taotao Wei

**Affiliations:** 1 National Laboratory of Biomacromolecules, Institute of Biophysics, Chinese Academy of Sciences, 100101 Beijing, China; 2 University of Chinese Academy of Sciences, 100049 Beijing, China; 3 Department of Pharmacology and Neuroscience, Creighton University School of Medicine, Omaha, NE 68178 USA

Correction to: *Cell Death & Disease* 10.1038/s41419-020-02937-5, published online 09 September 2020

During a recent review of the work, we noticed an error in Figure 1C (right panel). Specifically, two representative images corresponding to the 4-day and 6-day culture conditions were inadvertently misplaced during figure preparation. This error occurred due to an unintentional mix-up of datasets during the final figure assembly, where multiple image files with similar labeling were handled simultaneously. The corrected version of Figure 1C is now provided. This correction does not affect the results, interpretation, or conclusions of the study.

**Original Figure 1C** 
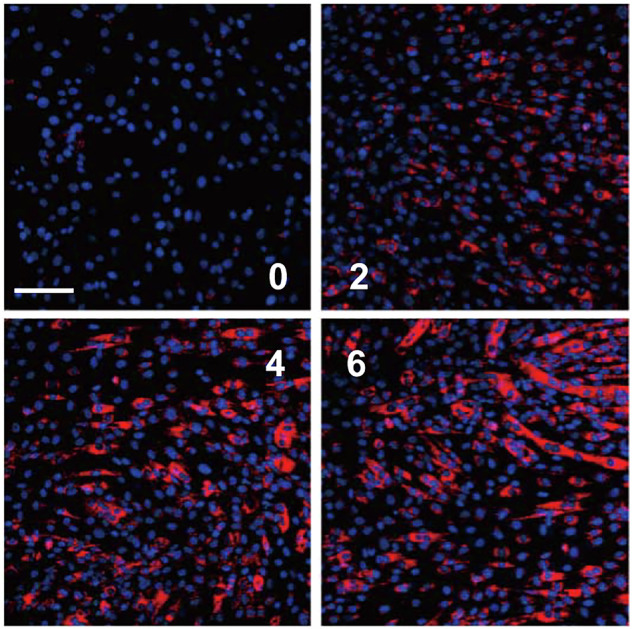


**Corrected Figure 1c** 
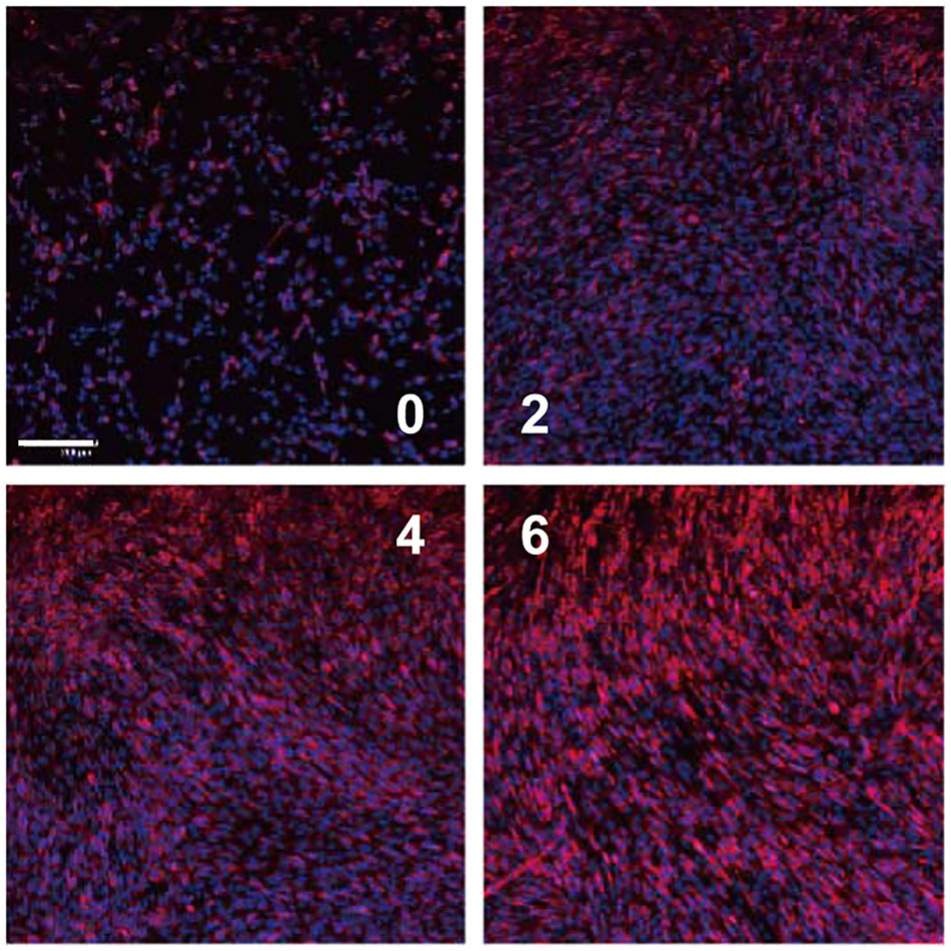


The original article has been corrected.

