# Supplementary material for: KAP1-associated transcriptional inhibitory complex regulates C2C12 myoblasts differentiation and mitochondrial biogenesis via miR-133a repression
Source: Cell Death Dis. 2020 Sep 9;11(9):732. doi: 10.1038/s41419-020-02937-5 (PMC7481787; doi:10.1038/s41419-020-02937-5)
Supplement: Supplementary file 1 — Supplementary Information 1 [file 41419_2020_2937_MOESM1_ESM.docx]

**Supplementary Information**

**Supplementary Information 1. Supplementary figures.**

**
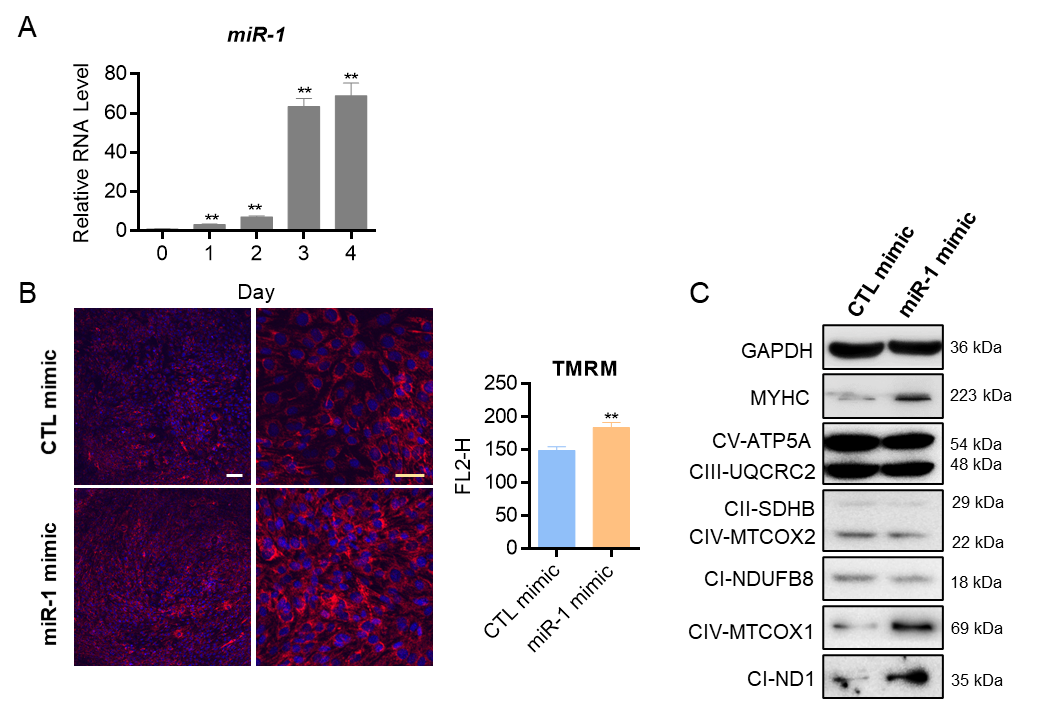
**

**Figure S1. miR-1 regulates the C2C12 differentiation and mitochondrial biogenesis. A.** RT-PCR quantification of miR-1 during cell differentiation. RNU6B was used as the control. Data are presented as the mean ± SEM (n = 4), ***P* < 0.01. **B.** miR-1 increases the mitochondrial transmembrane potential in C2C12 cells. C2C12 cells were transfected with miR-1 mimic for 72 h and stained with TMRM and Hochest. Intracellular TMRM fluorescence was measured by both FV-1000 microscope (left; scale bar: 100 μm and 50 μm) and flow cytometry (right). Data are presented as the mean ± SEM (n = 4), ***P* < 0.01. **C.** miR-1 mimic upregulates the expression levels of MYHC and mitochondrial complex subunits COX1 and ND1 in C2C12 cells. C2C12 cells were transfected with miR-1 mimic for 72 h and the expression levels of MYHC and mitochondrial respiratory complex subunits were detected by Western Blot.


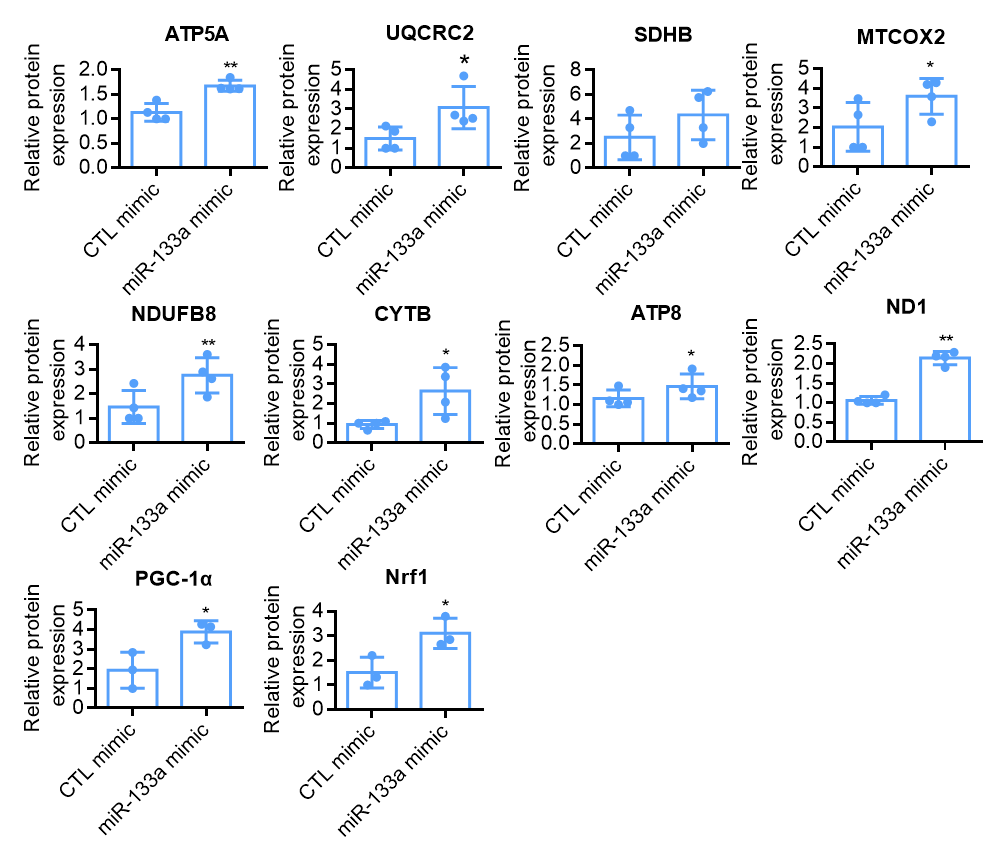


**Figure S2. Quantification of western blots shown in Figure 5B.** Data are normalized with GAPDH and presented as the mean ± SEM (n ≥ 3), **P* < 0.05, ***P* < 0.01.


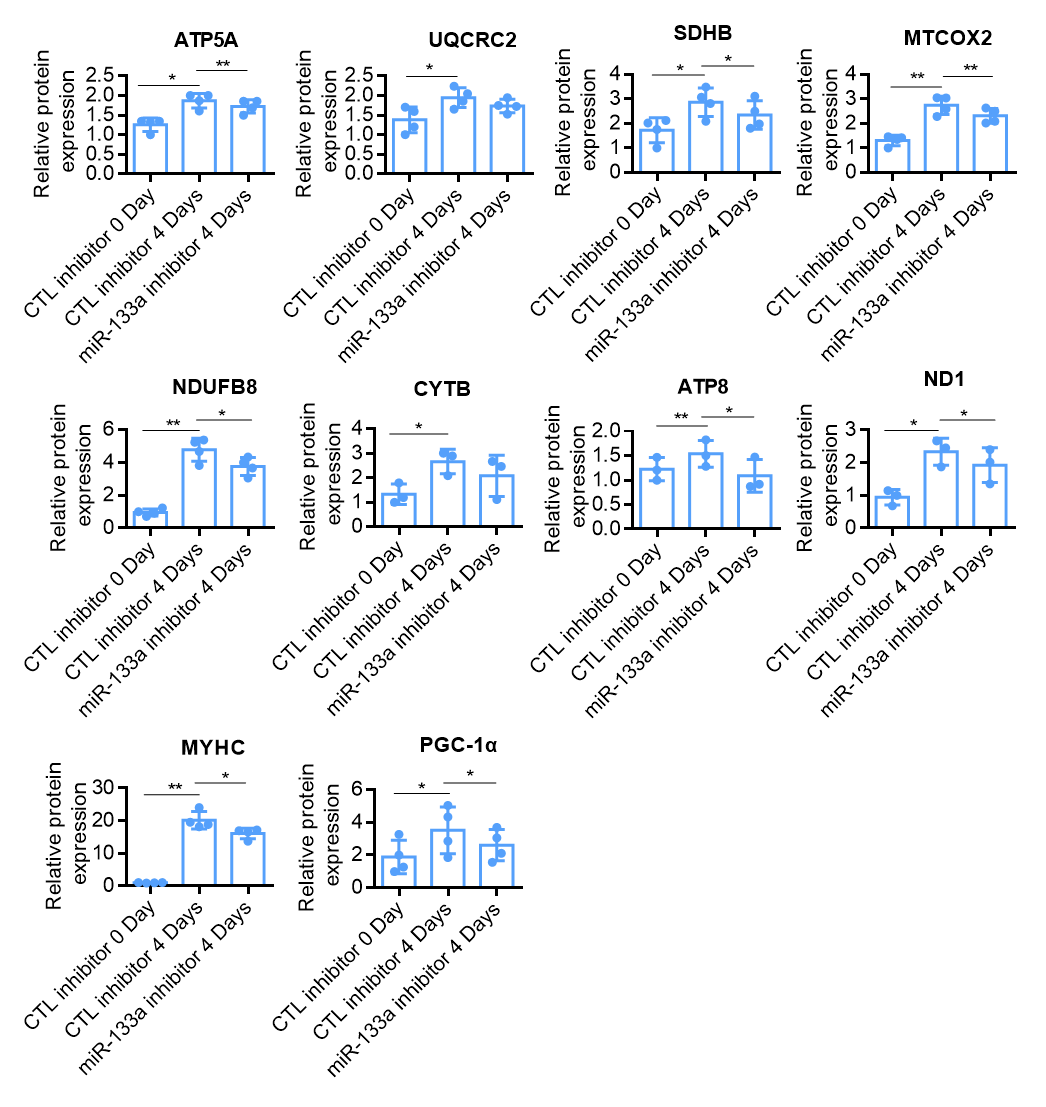


**Figure S3. Quantification of western blots shown in Figure 5C.** Data are normalized with GAPDH and presented as the mean ± SEM (n ≥ 3), **P* < 0.05, ***P* < 0.01.
