# Supplementary material for: KAP1-associated transcriptional inhibitory complex regulates C2C12 myoblasts differentiation and mitochondrial biogenesis via miR-133a repression
Source: Cell Death Dis. 2020 Sep 9;11(9):732. doi: 10.1038/s41419-020-02937-5 (PMC7481787; doi:10.1038/s41419-020-02937-5)
Supplement: Supplementary file 2 — Supplementary Information 2 [file 41419_2020_2937_MOESM2_ESM.docx]

**Supplementary Information**

**Supplementary Information 2. List for primers.**

Primers for SYBR green RT-PCR (mouse):

*Myhc* Forward: ACCGAAGGCGGAACTACTGTAAC

Reverse: ATCCAGGCTGCATAACGCTCT

*18S* Forward: GTAACCCGTTGAACCCCATT

Reverse: CCATCCAATCGGTAGTAGCG

*Myog* Forward: CGGTGGAGGATATGTCTGTTG

Reverse: GGTGTTAGCCTTATGTGAATGG

*Myod* Forward: CGCCATCCGCTATATCGAGG

Reverse: CTGTAGTCCATCATGCCGTCG

*Ckm* Forward: CTGACCCCTGACCTCTACAAT

Reverse: CATGGCGGTCCTGGATGAT

*Cox1* Forward: CCTATCACCCTTGCCATCAT

Reverse: GAGGCTGTTGCTTGTGTGAC

*Pecam* Forward: ATGGAAAGCCTGCCATCATG

Reverse: TCCTTGTTGTTCAGCATCAC

*Kap1* Forward: CGGCGCTATGGTGGATTGT

Reverse: GGTTAGCATCCTGGGAATCAGAA

Primers for Chip-PCR (mouse):

*miR-133a-F*: CTGATTTTGTTGCTTCCAAGTTCAGC

*miR-133a-R*: GCATCTGATAGTGTCAGCATCTGTTATC

*Gapdh-F*: GGGAAGGAAATGAATGAACCGCCG

*Gapdh-R*: CTAGAATACGCATTATGCCCGAGGAC

Primers for Taqman RT-PCR (mouse):

*miR-133a-3p* Forward: GCCGtttggtccccttcaac

Stem loop: GTCGTATCCAGTGCAGGGTCCGAGGTATTCGCAC

TGGATACGACcagctg

*miR-1-3p* Forward: GCCGCCtggaatgtaaagaagt

Stem loop: GTCGTATCCAGTGCAGGGTCCGAGGTA

TTCGCACTGGATACGACatacat

*Rnu6-1* Forward: ggatgacacgcaaattcgtgaagc

Stem loop: GTCGTATCCAGTGCAGGGTCCGAGGTATTCGCAC

TGGATACGACaaaatatggaac

Universal stem loop reverse: CAGTGCAGGGTCCGAGGT

Taqman probe: 5’-FAM-TCGCACTGGATACG-MGBNFQ-3’
