# Supplementary material for: KAP1-associated transcriptional inhibitory complex regulates C2C12 myoblasts differentiation and mitochondrial biogenesis via miR-133a repression
Source: Cell Death Dis. 2020 Sep 9;11(9):732. doi: 10.1038/s41419-020-02937-5 (PMC7481787; doi:10.1038/s41419-020-02937-5)
Supplement: Supplementary file 3 — Supplementary Information 3 [file 41419_2020_2937_MOESM3_ESM.docx]

**Supplementary Information**

**Supplementary Information 3. Details of fluorescence probes, antibodies and small RNAs.**

**Fluorescence probes:**

MitoTracker Red FM M22425, Invitrogen (Eugene, OR, USA): Working concentration was 100 nM, incubation at 37 °C for 30 min, captured by FV1000 or SIM DV (Delta Vision) OMX.

Hoechest 33342 C1022, Beyotime (Songjiang, Shanghai, China): Cell was treated with Hoechest at 37 °C for 10 min, and images were captured by FV1000.

DAPI D9542, Sigma-Aldrich (St. Louis, MO, USA): Fixed the cells with 4% paraformaldehyde at room temperature for 20 min, stained with DAPI for 10 min, then captured by FV1000.

MHC MF20, DSHB (Lowa, Lowa, USA): Cells were fixed with 4% paraformaldehyde at room temperature for 20 min, permeated with 0.2% Triton X-100 for 5 min, sealed with 5% BSA for 30 min, then applied with primary antibody overnight. Subsequently, cells were applied with secondary antibody for 1 h, washed twice with PBS, sealed with mounting medium, and photographed by FV1000.

H2DCFDA C6827, Invitrogen (Eugene, OR, USA): Cells were treated with 5 μM final concentration of H2DCFDA at 37 °C for 30 min, ROS level of 30,000 cells was measured by flow cytometer BD FacsCalibur.

**Antibodies:**

Cocktail ab110411 Abcam (Cambridge, Cambridgeshire, UK)

CYTB 55090-1-AP Proteintech (Chicago, IL, USA)

ATP8 26723-1-AP Proteintech (Chicago, IL, USA)

ND1 19703-1-AP Proteintech (Chicago, IL, USA)

COX1 13393-1-AP Proteintech (Chicago, IL, USA)

PGC-1α D162014-0025 Sangon Biotech (Shanghai, China)

Nrf1 12482-1-AP Proteintech (Chicago, IL, USA)

Kap1 15202-1-AP Proteintech (Chicago, IL, USA)

HP1 2616S CST (Danvers, MA, USA)

CHD4 14173-1-AP Proteintech (Chicago, IL, USA)

HDAC2 ab32117 Abcam (Cambridge, Cambridgeshire, UK)

GAPDH 60004-1-lg-100uL Proteintech (Chicago, IL, USA)

Flag D110005-0100 BBI (Crumlin, South Wales, UK)

V5 CW0094S CWBIO (Changping, Beijing, China)

Mfn1 13798-1-AP Proteintech (Chicago, IL, USA)

Mfn2 12186-1-AP Proteintech (Chicago, IL, USA)

Drp1 8579S CST (Danvers, MA, USA)

MHC MF20 DSHB (Lowa, Lowa, USA)

**Small RNAs:**

Kap1 siRNA (si-m-Trim28-001: siB150601094314; si-m-trim28-002: siG150911044503-1-5; siR NC: [siN0000001-1-10](https://www.ribobio.com/view_product.php?sku=siN0000001-1-10))

miR-mimics (micrON mmu-miR-133a-3p mimic: mir10000145-1-5; micrON mmu-miR-1b-3p mimic: [miR11458120451-1-5](https://www.ribobio.com/view_product.php?sku=miR11458120451-1-5); mimic negative control: miR01101-1-5)

miR-inhibitors (micrOFF mmu-miR-133a-3p inhibitor: miR20000145-1-5; inhibitor. negative control: miR02101-1-5) RiboBio (Guangzhou, China).
